# Radical Prostatectomy in Multimodal Setting: Current Role of Neoadjuvant and Adjuvant Hormonal or Chemotherapy-Based Treatments

**DOI:** 10.3390/curroncol32020092

**Published:** 2025-02-07

**Authors:** Marco Oderda, Giorgio Calleris, Giuseppe Carlo Iorio, Giuseppe Simone, Paolo Gontero

**Affiliations:** 1 Division of Urology, Department of Surgical Sciences, Molinette Hospital, University of Turin, 10126 Torino, Italy; giorgio.calleris@unito.it (G.C.); paolo.gontero@unito.it (P.G.); 2 Department of Oncology, Radiation Oncology, University of Turin, 10124 Torino, Italy; giorio@cittadellasalute.to.it; 3 Department of Urology, IRCCS “Regina Elena” National Cancer Institute, 00144 Rome, Italy; giuseppe.simone@ifo.it

**Keywords:** prostatectomy, prostate cancer, high risk, neoadjuvant, adjuvant, ADT, hormonal therapy, ARSI, docetaxel

## Abstract

The role of neoadjuvant and adjuvant hormonal or chemotherapy-based treatments before or after radical prostatectomy in localized or locally advanced high-risk prostate cancer (PCa) is currently debatable. European guidelines recommend adjuvant androgen deprivation therapy (ADT) only in pN1 patients after extended pelvic lymph node dissection based on outdated evidence on standard hormonal agents. The introduction of new-generation androgen receptor targeting agents (ARTAs) has revolutionized the treatment of metastatic PCa and might also impact the perioperative management of patients with high-risk localized disease. In the last years, a renewed interest has also arisen in chemotherapy-based neoadjuvant or adjuvant treatments alone or in combination with ADT and/or ARTAs. In the present review, we gathered the current evidence on the oncological outcomes of neoadjuvant and adjuvant systemic treatments in surgically treated patients with localized or locally advanced PCa. Despite mild benefits in terms of pathologic responses or oncological outcomes reported in some studies investigating ADT and/or chemotherapy in this setting of patients, strong evidence to support their use in clinical practice is lacking. Promising data in favor of ARTAs have been gathered from phase II trials and prospective series, but definitive results from phase III trials are awaited to confirm these findings.

## 1. Introduction

Around 22.5% of newly diagnosed prostate cancer (PCa) is represented by localized or locally advanced high-risk disease, according to a recent analysis of the Surveillance, Epidemiology and End Results (SEER) registries database (2010–2015) [1]. This subgroup has been shown to be life-threatening, with elevated all-cause and cancer-specific mortality rates, even in the elderly population [2,3], and it needs to be treated aggressively, possibly in a multimodal setting. In surgically treated patients, though, the role of neoadjuvant and adjuvant treatments remains highly debated.

Neoadjuvant treatments are commonly used in several types of cancers, including bladder, breast, and rectum. Nevertheless, current PCa guidelines of the European Association of Urology (EAU) [4] strongly recommend not to offer neoadjuvant androgen deprivation therapy (ADT) before surgery based on historical trials that investigated the role of LHRH agonists and first-generation anti-androgens, failing to show survival benefits in the neoadjuvant setting [5]. However, these trials enrolled mostly low-risk patients, had limited follow-up, and did not systematically evaluate pathological response. In the last years, several trials have been conducted to evaluate the role of new-generation androgen receptor targeting agents (ARTAs) as neoadjuvant treatments before surgery specifically in the high-risk population with adequate central pathological revision and long-term follow-up. These drugs showed a tremendous benefit in the treatment of metastatic disease and might revolutionize the perioperative management of high-risk patients.

As for adjuvant treatments, current EAU guidelines discourage prescribing ADT to pN0 patients, irrespective of the risk class [4]. Adjuvant ADT can be considered only in pN1 patients after extended pelvic lymph node dissection, mainly based on the results of the Messing trial that randomized N1 patients to immediate ADT versus observation, demonstrating an improvement in overall (OS), cancer-specific (CSS), and progression-free survival (PFS). It has to be noted that this trial was conceived and begun before the implementation of PSA in clinical practice and was underpowered, having enrolled less than half of target patients [6]. In the last years, several trials have evaluated the role of ADT, chemo-hormonal treatment, and ARTAs in the adjuvant setting. To date, however, no consensus has been reached.

This review of the literature aims to gather the current evidence on the oncological outcomes of neoadjuvant and adjuvant hormonal or chemotherapy-based treatments before or after radical prostatectomy.

## 2. Methods

A comprehensive narrative review of the literature was performed using PubMed and Scopus databases, using MeSH terms “prostate cancer”, “radical prostatectomy”, “hormonal therapy”, “chemotherapy”, “neoadjuvant”, and “adjuvant” in different combinations with the Boolean operators “AND/OR”, limiting the search to original studies published in English within the last 10 years. Publications relevant to the search and their cited references were retrieved and evaluated independently by 2 authors (M.O., G.C.) for inclusion in the review. We included only prospective studies (randomized or single-arm) that evaluated the role of neoadjuvant or adjuvant hormonal or chemo-hormonal treatments before or after RP. Studies focusing on radiation therapy were excluded. The initial search found 316 results; after exclusion of unsuitable papers for different reasons, 25 studies were included in the review.

## 3. Evidence Synthesis

### 3.1. Neoadjuvant Hormonal Therapy

In the last 10 years, ten studies were found addressing the role of neoadjuvant ADT, of which seven were phase II randomized controlled trials (RCTs) and three were phase II single-arm series (Table 1). All studies enrolled high-risk patients, and some of them included a certain amount of intermediate-risk patients.

Only one of these studies investigated “standard” ADT alone, evaluating whether preoperative Degarelix influenced surrogates of disease control; patients receiving Degarelix had increased levels of intratumoral dihydrotestosterone and lower serum levels of FSH and inhibin B, but no differences were found in terms of postoperative positive margins (PSMs) [7].

All other studies investigated ARTAs, alone or in different combinations, demonstrating promising results in terms of both pathological complete response (pCR, intended as absence of residual tumor at histological examination) and minimal residual disease (MRD, defined as residual tumor burden ≤5 mm at cross-sectional slice). The rates of pCR and MRD were as high as 14% and 38%, respectively, in two studies that tested apalutamide + ADT [8,9]. In a pooled analysis of three RCTs that evaluated intense neoadjuvant deprivation with abiraterone and/or enzalutamide prior to radical prostatectomy, McKay et al. identified a strong correlation between pathological responses and biochemical recurrence (BCR); at a median follow-up of 3.6 years, only two patients with a pathological response experienced BCR, and no patients with an exceptional pathological response developed metastases [10].

ARTA monotherapy without the use of ADT did not achieve good pathological responses in two phase II trials. The NEAR trial tested a 12-week course of neoadjuvant apalutamide monotherapy prior to radical prostatectomy in the treatment of 30 intermediate- and high-risk PCa without observing any pCR [11]. In a phase II RCT, enzalutamide alone was inferior to the combination of enzalutamide, dutasteride, and LHRH analogue in terms of PSM, pCR, and MRD [12]. The combination of different ARTAs in a super-intensification protocol was evaluated by two phase II trials. In 2019, McKay et al. randomized 75 patients to receive neoadjuvant enzalutamide and leuprolide with or without abiraterone and prednisone, finding a trend towards improved pathologic outcomes with the ARTA combination, although it was not statistically significant [13]. In 2021, 118 high-risk patients were randomized to abiraterone, prednisone, and leuprolide with or without apalutamide; the neoadjuvant treatment resulted in favorable pathologic responses in 21% of patients, but responses were similar between the two arms [14].

The duration of neoadjuvant treatments was not homogeneous across studies, ranging from 3 to 6 months. The only study that compared different treatment durations was performed in 2014 by Taplin et al., finding higher rates of pCR and MRD in patients receiving neoadjuvant LHRH analogues plus abiraterone administered for 24 weeks rather than 12 weeks [15].

To date, data on survival outcomes are scarce; three-year recurrence-free survival (RFS) was not significantly different between patients treated with abiraterone plus leuprolide and leuprolide (75%) and leuprolide alone (71%) followed by prostatectomy [16]. The NEAR trial found a 2-yr BCR-free survival of 86% in 30 patients treated with a 12-week course of neoadjuvant apalutamide before prostatectomy [11]. Finally, a 1-yr PFS of 90% was found in 30 patients treated for 6 months with ADT and darolutamide prior to surgery [17].

**Table 1:** Main studies on neoadjuvant hormonal treatments before prostatectomy.

Author, Year	Therapy	Design	Arms of Neoadjuvant	Positive Margins	pCR	MRD	pCR + MRD	Survival
Taplin, 2014 [15]	ABI	Phase II RCT, 58 HR men	12-w ABI + L vs. 24-w ABI + L	19% vs. 10% (pNR)	4% vs. 10% (pNR)	0% vs. 14% (pNR)	4% vs. 24% (pNR)	-
Montgomery, 2017 [12]	ENZA	Phase II RCT, 48 IR-HR men	6-mo ENZA vs. 6-mo ENZA + dutasteride + L	16% vs. 22% (pNR)	0% vs. 4% (pNR)	0% vs. 13% (pNR)	0% vs. 17% (pNR)	-
Sayyid, 2017 [7]	Degarelix	Phase II RCT, 39 HR men	3-mo Degarelix vs. 3-mo Degarelix + bicalutamide vs. 3-mo LHRHa + bicalutamide	38% vs. 21% vs. 33% (*p* 0.61)	-	-	-	-
Estathiou, 2019 [16]	ABI	Phase II RCT, 58 HR men	3-mo L vs. 3-mo ABI + L	14% vs. 5% (*p* 0.17)	-	-	-	71% vs. 75% 3-yr RFS (*p* 0.28)
McKay, 2019 [13]	ABI + ENZA	Phase II RCT, 75 IR-HR men	24-w ENZA + L vs. 24-w ABI + ENZA + L	12% vs. 18% (pNR)	8% vs. 10% (pNR)	8% vs. 20% (pNR)	16% vs. 30% (*p* 0.2)	-
McKay, 2021 [14]	ABI + APA	Phase II RCT, 118 IR-HR men	6-mo ABI + L vs. 6-mo ABI + APA + L	12% vs. 7% (pNR)	10% vs. 13% (pNR)	10% vs. 9% (pNR)	20% vs. 22% (*p* 0.4)	-
Lee, 2022 (NEAR trial) [11]	APA	Phase II single-arm, 30 IR-HR men	12-w APA	16%	0%	NR	NR	86% 2-yr bRFS
Devos, 2023 (ARNEO trial) [9]	APA+ Degarelix	Phase II RCT, 89 HR men	3-mo Degarelix + APA vs. 3-mo Degarelix + placebo	18% vs. 18% (pNR)	0% vs. 0% (pNR)	38% vs. 9% (*p* 0.002)	38% vs. 9% (*p* 0.002)	-
Wei, 2023 [8]	APA	Phase II single-arm, 7 advanced PCa	4-mo APA + ADT	-	14%	-	-	-
Cardili [18]	ABI + APA	Phase II RCT, 62 HR men	3-mo ADT + ABI + APA vs. 3-mo ADT + ABI	32%	-	-	22.5%	-
Zhuang, 2024 [17]	DARO	Phase II single-arm, 30 HR men	6-mo DARO + ADT	13%	7%	33%	40%	90% 1-yr PFS

Legend: ABI: abiraterone; ADT: androgen deprivation therapy; APA: apalutamide; bRFS: biochemical recurrence-free survival; DARO: darolutamide; ENZA: enzalutamide; HR: high-risk; IR: intermediate-risk; L: leuproreline; LHRHa: LHRH analogue; MRD: minimal residual disease; NR: not reported; p NR: p not reported; pCR: pathologic complete response; PFS: progression-free survival; RCT: randomized controlled trial; RFS: recurrence-free survival.

### 3.2. Neoadjuvant Chemo-Hormonal Therapy

A renewed interest in neoadjuvant chemo-hormonal treatments rose after the results of the PUNCH trial, a phase III RCT that evaluated the role of preoperative docetaxel plus ADT versus surgery upfront [19]. The authors randomized 788 patients, 738 of which ultimately underwent prostatectomy. Even though the primary study endpoint, 3-yr biochemical progression-free survival (bPFS), was not met, there was an improvement in metastasis-free survival (HR 0.70, 95%CI 0.51–0.95) and OS (HR 0.61, 95%CI 0.40–0.94) for the patients in the neoadjuvant arm. At 10 years, the survival probabilities were 0.74 (95%CI 0.67–0.83) and 0.80 (95%CI 0.72–0.88). In 2021, Chi et al. investigated the effectiveness of neoadjuvant chemo-hormonal therapy versus surgery upfront in oligometastatic patients, performing a propensity-score match on a subgroup of 34 cases with a control arm represented by ADT only [20]. Complete and partial responses were seen in 11% and 23% of patients in the neoadjuvant group, respectively. PFS was higher and PSMs were lower in patients receiving prior chemo-hormonal therapy, whereas toxicities were reported as acceptable. When abiraterone was added to ADT, no benefits were demonstrated with the addition of docetaxel [21] or cabazitaxel [22] in terms of PSM, pCR, MRD, or survival outcomes.

More recently, Qian et al. randomized 141 cases with locally advanced PCa to neoadjuvant hormonal therapy (ADT + bicalutamide, without intensification) versus neoadjuvant docetaxel-based chemo-hormonal therapy [23]. Their findings showed that patients who underwent prostatectomy after prior chemo-hormonal therapy had significant benefits in 3-yrs BCR-free survival (29% vs. 9.5%, *p* 0.002) but none in terms of pathological downstaging and MRD.

Main studies on neoadjuvant chemotherapy before prostatectomy, with or without ADT, are summarized in Table 2.

### 3.3. Adjuvant Systemic Treatments

Current guidelines recommend adjuvant ADT in pN1 patients [4] after extended pelvic lymph node dissection, following the well-known RCT by Messing et al. [6] that randomized 98 men with node-positive PCa to receive immediate ADT or to be observed, finding a significant improvement in OS (hazard ratio 1.84 (95%CI 1.01–3.35), *p* 0.04), CSS (hazard ratio 4.09 (95%CI 1.76–9.49), *p* 0.0004), and PFS (hazard ratio 3.42 (95% CI 1.96–5.98), *p* < 0.0001). In cases pN0, instead, adjuvant treatment is not recommended [4], irrespective of the risk category. Several trials have recently investigated the role of adjuvant treatments, including ADT and chemotherapy, but no conclusive findings have emerged.

In 2016, Chang et al. randomized 209 patients with high-risk, localized PCa to adjuvant complete androgen blockade (CAB) or bicalutamide 150 mg daily, finding a longer biochemical RFS in the CAB group (*p* 0.004) at a median follow-up of 27 months, with similar side-effects [26]. In 2019, a prospective, observational, Chinese study explored the use of adjuvant ADT after surgery (physician’s choice) on 189 patients with high-risk localized and locally advanced PCa [27]. Most patients (66.1%) received CAB as adjuvant ADT for a median duration of 20 months. A non-significant trend in reduction of biochemical RFS was noted in the CAB group (15%) compared to LHRH agonists (16%) and antiandrogens (19%).

Two trials evaluated the role of ARTAs as adjuvant treatments. In 2023, McKay et al. randomized 82 patients with localized, unfavorable intermediate-risk and high-risk PCa, previously treated with 6-mo neoadjuvant treatment, to receive either apalutamide, abiraterone, prednisone, plus leuprolide (AAPL) for 12 months, or observation, with a non-statistically significant benefit in 3-year biochemical PFS for AAPL group (81% versus 72%, hazard ratio, 0.81; 90%CI 0.43–1.49) [28]. However, the study was underpowered to detect differences between arms due to insufficient recruitment. More recently, Shore et al. investigated the role of 12-months adjuvant ADT + apalutamide after surgery in 108 men with high-risk localized PCa, most of them having Grade Group 5 disease. The confirmed 2-yrs biochemical RFS was 100%, with a 77% testosterone recovery rate at 12 months post treatment [29].

As for adjuvant chemotherapy, no benefit was found from docetaxel given after prostatectomy compared to surveillance in an open-label RCT on 459 men with high-risk PCa [30]. The same conclusions were drawn by Lin et al. in the Veterans Affairs Cooperative Studies Program study #553, an RCT that failed to demonstrate statistically significant improvement in PFS for the intention-to-treat population as a whole in 298 patients at high risk for relapse after prostatectomy [31]. In 2018, an RCT evaluated the role of mitoxantrone and prednisone added to ADT as adjuvant treatment after prostatectomy, but the results were again negative; mitoxantrone did not improve OS, while increased deaths from other malignancies were found at a median follow-up of 11 years (36% versus 18% in the ADT alone arm) [32].

Interestingly, all of these studies included only a minority of pN1 patients, ranging from 6% to 17%. Main studies evaluating adjuvant treatments after prostatectomy are summarized in Table 3.

## 4. Discussion

The role of neoadjuvant and adjuvant hormonal or chemotherapy-based treatments before or after radical prostatectomy remains debatable. This is mainly due to the scarce efficacy of standard ADT, which failed to show significant benefits in men with high-risk, localized, or locally advanced PCa. The only trial that demonstrated significant improvements in OS, CSS, and PFS is the well-known Messing trial [6], conducted with 98 pN1 patients, which strongly influenced the EAU guidelines [4] despite being conceived in the era pre-PSA and underpowered due to scarce accrual. Currently, however, pN1 men usually undergo a risk-adapted strategy, with those at lower risk avoiding, at least initially, adjuvant ADT and its side effects or whole pelvis radiotherapy, for which the indications remain unclear [33,34,35]. Indeed, results of ongoing trials involving radiotherapy in the pN1 setting are eagerly awaited (NRG-GU008 [36], ANZUP1801 [37]).

As for chemotherapy, no tangible advantages have been demonstrated with the use of docetaxel or other agents in the neoadjuvant or adjuvant setting. A renewed interest in neoadjuvant chemo-hormonal therapy arose after the results of the PUNCH trial [19] showed an improvement in metastasis-free survival and OS for patients in the neoadjuvant arm. However, as stated by the authors, the overall number of deaths in the study was low, and cancer-specific deaths were even lower. In addition, the restricted mean survival benefit of 4 months at 10 years was only marginal. The PUNCH trial failed to meet its primary endpoint, 3-yr BCR-free survival; instead, significant benefits in 3-yr BCR-free survival were shown by Qian et al. in favor of the chemotherapy arm [23]. Other trials evaluating neoadjuvant chemotherapy did show modest benefits in terms of pCR or MRD, but survival outcomes were hardly affected. Furthermore, when an ARTA was introduced as a comparator arm, the addition of chemotherapy did not add any further benefits in terms of pathological or survival outcomes.

The introduction of ARTAs has revolutionized the management of advanced PCa, with a profile of efficacy and tolerability superior to chemotherapy. The anticipation of ARTAs in the localized or locally advanced high-risk setting as neoadjuvant or adjuvant treatments could be key to improving the outcomes of patients undergoing radical prostatectomy in a multimodal approach aiming to treat occult metastatic disease. The results of STAMPEDE and PEACE-1 trials supported the adoption of prostate radiotherapy even in oligometastatic cases, together with hormonal treatments [38,39]; we can hypothesize that these findings can be replicated for surgery, together with the use of ARTAs.

To date, small, phase II trials have demonstrated only partial benefits with the use of ARTAs in the neoadjuvant setting in terms of pCR, MRD, or bRFS. In the adjuvant setting, the most interesting data come from the prospective study of Shore et al., which reported a 100% 2-yr biochemical RFS in high-risk patients undergoing surgery followed by ADT plus apalutamide [29]. An advantage of ARTAs administration compared to chemotherapy is the ease of administration and the good profile of tolerability, even though a systematic review and meta-analysis found that cardiovascular and thrombo-embolic adverse events associated with the use of ADT plus ARTAs in the neoadjuvant setting in patients with localized PCa undergoing prostatectomy can occur in up to 4.6% of cases, therefore requiring careful assessment of thrombotic risk and prophylactic anticoagulants in this setting [40].

The results of several phase III trials are awaited in the next months. Among the others, we cite the PROTEUS trial that randomized patients with very high-risk PCa features to ADT plus apalutamide or placebo 6 months prior to and after prostatectomy and the GETUG-20 trial that assessed the role of 2 years of adjuvant leuprolide in patients with a Gleason score ≥ 7, pT3b disease, and post-operative PSA < 0.1 ng/mL. Furthermore, the STAPLE trial has recently started to recruit patients who are oligometastatic at PSMA PET to undergo prostatectomy followed by ADT versus ADT plus apalutamide, pushing the boundaries of surgery in a multimodal approach.

The limited evidence derived from the included studies, most of them being small, phase II trials, represents the main limitation of our work, together with the heterogeneity of these studies in terms of design, treatment arms, and schedule. Nonetheless, data presented in this study come almost exclusively from prospective RCTs.

## 5. Conclusions

Currently, the use neoadjuvant or adjuvant systemic treatments together with surgery is not recommended, except for ADT in pN1 patients. Despite mild benefits in terms of pathologic responses or oncological outcomes that have been reported in some studies investigating ADT and/or chemotherapy in high-risk, localized, or locally advanced PCa, strong evidence to support their use in clinical practice is lacking. Promising data in favor of ARTAs have been gathered from phase II trials and prospective series, but definitive results from phase III trials are awaited to confirm these findings.

## Figures and Tables

**Table 2:** Main studies on neoadjuvant chemotherapy before prostatectomy.

Author, Year	Therapy	Design	Arms of Neoadjuvant	Positive Margins	pCR	MRD	pCR + MRD	Survival
Silberstein, 2015 [24]	Estramustine	Phase II, 34 vs. 37 HR men	6-mo ADT + Estramustine + CP vs. surgery upfront	24% vs. 46% (pNR)	0% vs. 0% (pNR)	-	-	No difference in survival
Zhao, 2015 [25]	Docetaxel	Single arm, 28 locally advanced PCa	18–24-w Docetaxel	25%	0%	-	-	10-yr bRFS 33.5%
Eastham, 2020 [19]	Docetaxel	Phase III RCT, 738 HR men	18–24-w Docetaxel + LHRHa vs. surgery upfront	18% vs. 45% (*p* <0.001)	0% vs. 0% (pNR)	-	-	10-yr OS: HR 0.61 for NCH
Chi, 2021 [20]	Docetaxel	Propensity score matching, 130 oligometastatic men	18–24-w Docetaxel + LHRHa vs. surgery upfront (vs. ADT only)	18% vs. 47% (pNR)	12% vs. 0% (pNR)	23% vs. 29% (pNR)	35% vs. 29% (pNR)	PFS HR 0.11 (*p* 0.004)
Fleschner, 2022 [22]	Cabazitaxel	Phase II RCT, 77 HR men	24-w Cabazitaxel + ABI + L vs. 24-w ABI + L vs.	-	5% vs. 9% (pNR)	39% vs. 36% (*p* 1.0)	43% vs. 45% (pNR)	No difference in bRFS
Zhuang, 2023 [21]	Docetaxel	Pooled analysis of 2 RCTs, 137 HR men	24-w Docetaxel + ADT vs. 24-w ABI + ADT vs. 24-w ADT	21% vs. 19% vs. 24% (*p* 0.8)	17% vs. 19% vs. 0% (*p* 0.01)	11% vs. 13% vs. 2% (*p* 0.2)	28% vs. 32% vs. 2% (*p* 0.002)	3-yr bPFS 42% vs. 51% vs. 61%
Qian, 2024 [23]	Docetaxel	Phase II RCT, 141 HR men	24-w Docetaxel + ADT vs. 24-w ADT	22% vs. 21%	1% vs. 0% (*p* 0.1)	8% vs. 2% (*p* 0.3)	10% vs. 2% (*p* 0.3)	3-yr bPFS 29% vs. 9%

Legend: ABI: abiraterone; ADT: androgen deprivation therapy; bPFS: biochemical progression-free survival; bRFS: biochemical recurrence-free survival; CP: carboplatin + paclitaxel; HR: high-risk; L: leuproreline; LHRHa: LHRH analogue; MRD: minimal residual disease; NCH: neoadjuvant chemohormonal; NR: not reported; p NR: p not reported; pCR: pathologic complete response; PFS: progression-free survival; RCT: randomized controlled trial; RFS: recurrence-free survival.

**Table 3:** Main studies on adjuvant treatments after prostatectomy.

Author, Year	Therapy	Design	Arms of Neoadjuvant	pN+	bPFS	PFS	CSS	OS
Chang, 2016 (CU-1005 trial) [26]	ADT	Phase II RCT, 209 HR men	9-mo CAB vs. 9-mo bicalutamide 150 mg	20 (10%)	2-yr 80% vs. 63% (*p* 0.004)	-	-	-
Ahlgren, 2018 [30]	Docetaxel	Phase III RCT, 459 HR men	24-wk Docetaxel vs. observation	55 (12%)	5-yr 45% vs. 38%	5-yr 55% vs. 62%	5-yr 97% vs. 99%	5-yr 95% vs. 96%
Hussain, 2018 (SWOG S9921) [32]	Mitoxantrone	Phase III RCT, 961 HR men	ADT + Mitoxantrone vs. ADT	162 (17%)	-	-	10-yr 72% vs. 72%	10-yr 87% vs. 86%
Ye, 2019 [27]	ADT	Prospective, 189 HR/locally advanced PCa	ADT (length not specified)	23 (12%)	2-yr 82%	-	-	-
Lin, 2020 [31]	Docetaxel	Phase III RCT, 298 HR men	24-wk Docetaxel vs. observation	NR	-	47% vs. 53% (*p* 0.2)	-	92% vs. 89% (*p* 0.4)
McKay, 2023 [28]	ABI + APA	Phase II RCT, 82 IR/HR men	12-mo ABI + APA + L vs. observation	14 (17%)	4-yr: 67% vs. 61%	-	-	-
Shore, 2024 (NCT04523207) [29]	APA	Phase II single-arm, 108 HR men	12-mo APA + ADT	6 (6%)	2-yr 100%	-	-	-

Legend: ABI: abiraterone; ADT: androgen deprivation therapy; APA: apalutamide; bPFS: biochemical progression-free survival; CAB: complete androgen blockade; CSS: cancer-specific survival; HR: high-risk; IR: intermediate-risk; L: leuproreline; NR: not reported; OS: overall survival; PFS: progression-free survival; RCT: randomized controlled trial.
